# Folliculocystic and Collagen Hamartoma: A Subset of Fibrous Cephalic Plaque

**DOI:** 10.7759/cureus.14987

**Published:** 2021-05-12

**Authors:** Anita S Savell, Kyle Norton, Michael R Heaphy

**Affiliations:** 1 Dermatology, University of Nevada Reno School of Medicine, Reno, USA; 2 Dermatopathology, Skin Cancer and Dermatology Institute, Reno, USA

**Keywords:** tuberous sclerosis complex, fibrous cephalic plaque, folliculocystic and collagen hamartoma, hamartoma

## Abstract

Tuberous sclerosis complex is known to cause a variety of cutaneous hamartomas, most commonly hypomelanotic macules, angiofibromas, shagreen patches, and fibrous cephalic plaques. In recent years, a new cutaneous hamartoma that bears physical and histological resemblance to fibrous cephalic plaque has been proposed called folliculocystic and collagen hamartoma. The primary difference between the two diagnoses is the histologic presence of infundibular cysts in the latter. However, some authors have called into question if the two diagnoses are truly distinct. In this case report, we present a patient with tuberous sclerosis complex and fibrous cephalic plaque with infundibular cysts and propose that the presence of cysts should be incorporated into the possible histologic features of fibrous cephalic plaque.

## Introduction

Many people with tuberous sclerosis complex (TSC) have cutaneous manifestations: hypomelanotic macules (97.2%), facial angiofibromas (74.5%), shagreen patches (48%), fibrous cephalic plaques (FCPs) (18.9%), and periungual fibromas (15.1%) [1]. FCP typically presents as large, smooth-to-bumpy, firm-to-rubbery plaques on the forehead, face, scalp, or neck of patients with TSC [2]. It is histologically characterized by thick collagen deposition with perifollicular fibrosis and may demonstrate increased numbers of dilated vessels [2].

Folliculocystic and collagen hamartoma (FCCH) has been proposed as a new entity associated with TSC. It presents as a complex hamartoma with thick collagen deposition, concentric perifollicular fibrosis, and keratin-filled infundibular cysts [3]. On physical examination, the lesions appear as “large, painless, infiltrated plaques studded with follicular comedo-like openings and cysts containing and draining a keratinous or purulent material” [3] and have been reported on the face, scalp, abdomen, back, and thigh [4]. Since its initial characterization, 12 cases of FCCH have been reported, six of which were from the original report from Torrelo et al. in 2012 [3-9]. Here, we present a case of FCP with infundibular cysts on the scalp and forehead in a young man with a previously established diagnosis of TSC.

## Case presentation

**Figure 1:**
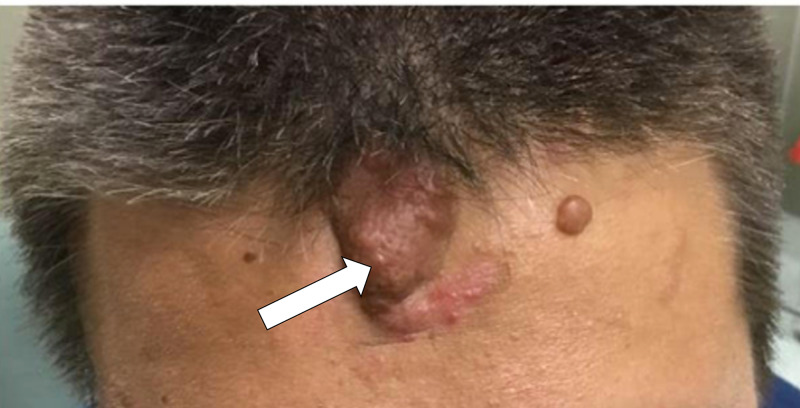
Clinical presentation of fibrous cephalic plaque on the forehead of a 17-year-old male. View of a large exophytic mass on the central forehead with prominent comedones (white arrow).

**Figure 2:**
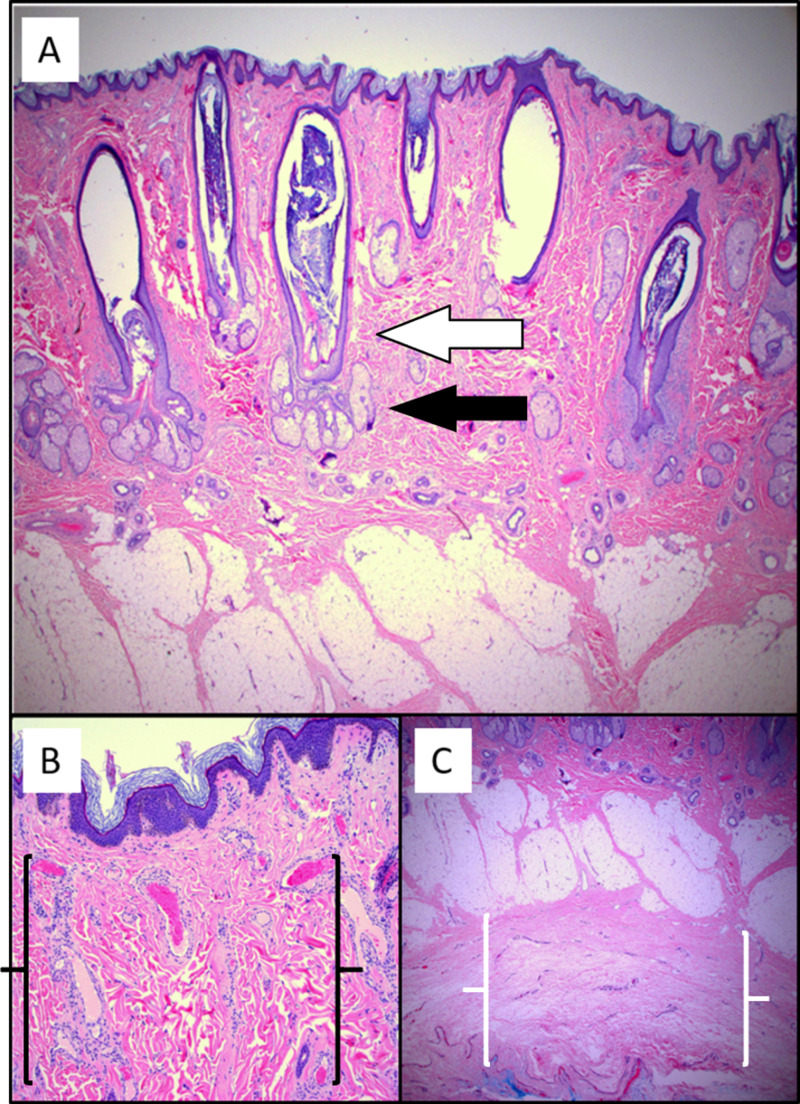
Photomicrograph of the exophytic forehead lesion stained with hematoxylin and eosin (A) Dilated, cystic hair follicles (white arrow) with hyperplastic sebaceous glands displaced to the lower portion of the dermis (black arrow). (B) Increased vascularity and fibrocytes (black brackets). (C) Expansion of subcutaneous collagen (white brackets) underlying normal adipose tissue.

A 17-year-old male with a known history of TSC and epilepsy presented to the dermatology clinic with multiple angiofibromas on the face and a large, painless exophytic mass on his central forehead. He requested excision due to cosmetic concerns (Figure 1). Microscopic examination of the lesion demonstrated epidermal papillomatosis, cystically dilated hair follicles (Figure 2A), hyperplastic sebaceous lobules displaced to the lower portions of the dermis by the cystic expansion of the follicular infundibulum (Figure 2A), and increased vascularity and fibrocytes (Figure 2B). There was also an expansion of the subcutaneous collagenous tissue which greatly contributed to the nodularity of the lesion clinically (Figure 2C).

**Figure 3:**
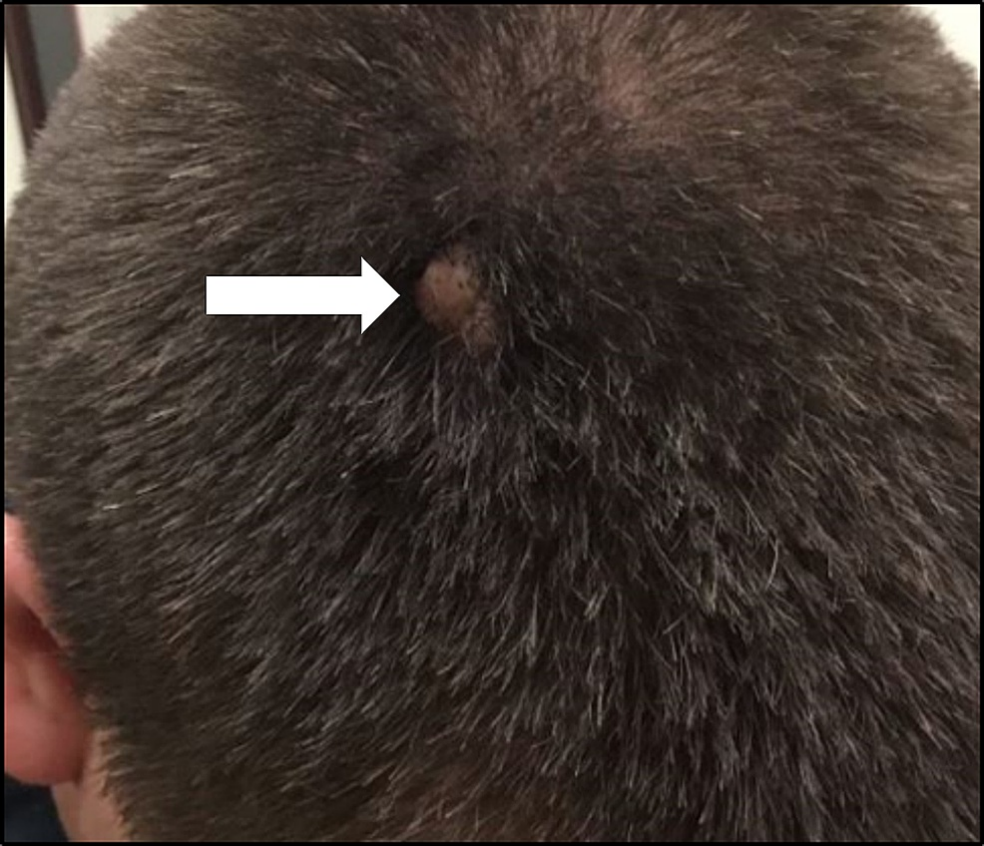
Clinical presentation of FCP with cyst formation previously confused for dissecting cellulitis on the scalp of a 17-year-old male. Nodular lesions previously excised from the patient's scalp (white arrow). FCP: fibrous cephalic plaque

Three previous excisions taken from similar nodules on the scalp showed follicular cysts which had ruptured and an associated granulomatous inflammatory reaction and perifollicular fibrosis (Figure 3). The nodules were initially diagnosed as dissecting cellulitis, but on review, at the time of subsequent biopsies, they were found to meet the criteria for a diagnosis of FCP with cyst formation. 

## Discussion

In 2012, FCCH was formally described as a new, diagnostically unique hamartoma to be associated with TSC. However, some authors have called into question whether FCCH is truly distinct from FCP due to their many clinical and histopathologic similarities [4]. Histologically, FCCH is distinguished from FCP by the presence of keratin-filled infundibular cysts [3]. On physical examination, FCP and FCCH may be difficult to distinguish, though FCCH has been reported to have comedones which may drain purulent material [3] and to be located on areas of the body not classic for FCP such as the back and thigh in approximately half of the reports [4].

The pathogenesis of cutaneous hamartomas in TSC is still being investigated. However, it has been suggested that angiofibroma, shagreen patch, ungual fibroma, and FCP develop as a result of mutations to fibroblasts [10]. It could be asserted that the presence of cysts within FCP does not change the fundamental defect in fibroblasts that cause the lesion, and therefore the addition of cysts does not warrant a distinct diagnosis.

There is an analogous example in dermatopathology where the fibroepithelial hamartomas of Birt-Hogg-Dube syndrome, fibrofolliculomas and trichodiscomas, are now accepted to represent the same entity with a spectrum of morphological features [11]. Both are regarded to be hamartomas of the fibrous sheath of the hair follicle, the distinguishing feature between trichodiscoma and fibrofolliculoma is the presence of thin strands of follicular epithelium in the latter. The selected designation may depend on what section one reviews through a single biopsy specimen. Trichodiscoma is now regarded to be a fibrofolliculoma in which the distinctive follicular epithelial strands are lacking [11].

We speculate that cysts may be a feature of FCP, and the presence of cysts should be incorporated into the list of possible features seen in FCP. As was described in our case, these cysts may rupture, making it difficult to establish the primary diagnosis due to obliteration of primary tissue architecture by granulomatous inflammation.

While FCP is not an optimal term to represent lesions outside of the cephalic distribution where some FCCH lesions have been reported, the presence of these lesions on non-cephalic, hair-bearing locations of the body alone does not warrant a distinct diagnosis. The diagnosis of TSC requires a combination of major and/or minor diagnostic features of which there are 11 and six, respectively (Table 1) [12].

**Table 1:** Major and minor diagnostic criteria for tuberous sclerosis complex.

Major Criteria	Minor Criteria
Hypomelanotic macules (3, at least 5-mm diameter)	“Confetti” skin lesions
Angiofibromas (3) or fibrous cephalic plaque	Dental enamel pits (3)
Ungual fibromas (2)	Intraoral fibromas (2)
Shagreen patch	Retinal achromic patch
Multiple retinal hamartomas	Multiple renal cysts
Cortical dysplasias	Nonrenal hamartomas
Subependymal nodules	
Subependymal giant cell astrocytoma	
Cardiac rhabdomyoma	
Lymphangioleiomyomatosis (LAM)	
Angiomyolipomas	

Importantly, FCP is a well-known major diagnostic feature of TSC. Because FCP is a more well-established manifestation of TSC, we suggest that lesions that meet the diagnostic criteria for FCP or FCCH simply be described as FCP.

## Conclusions

Here, we presented a case of FCP with histologic findings of infundibular cysts in a patient with known TSC. To further complicate the diagnosis of TSC with a separate entity called FCCH simply due to the histologic presence of cysts is unnecessary. Analogous examples exist in dermatopathology in the context of the hamartomas trichodiscoma and fibrofolliculoma in Birt-Hogg-Dube syndrome. In conclusion, we see no reason to complicate the spectrum of cutaneous hamartomas with a new entity called FCCH.
